# Altered regional gray matter in Congenital Adrenal Hyperplasia (CAH)

**DOI:** 10.1016/j.yhbeh.2025.105766

**Published:** 2025-06-04

**Authors:** Eileen Luders, Debra Spencer, Christian Gaser, Ajay Thankamony, Ieuan Hughes, Umasuthan Srirangalingam, Helena Gleeson, Melissa Hines, Florian Kurth

**Affiliations:** a Department of Women’s and Children’s Health, Uppsala University, Uppsala 75237, Sweden; b Swedish Collegium for Advanced Study (SCAS), Uppsala 75238, Sweden; c School of Psychology, University of Auckland, Auckland 1010, New Zealand; d Laboratory of Neuro Imaging, School of Medicine, University of Southern California, Los Angeles 90033, USA; e Department of Psychology, University of Cambridge, Cambridge CB23RQ, UK; f Department of Neurology, Jena University Hospital, Jena 07747, Germany; g Department of Psychiatry and Psychotherapy, Jena University Hospital, Jena 07747, Germany; h German Center for Mental Health (DZPG), Germany; i Department of Paediatrics, Addenbrooke’s Hospital, University of Cambridge, Cambridge CB20QQ, UK; j Weston Centre for Paediatric Endocrinology & Diabetes, Addenbrooke’s Hospital, University of Cambridge, Cambridge CB20QQ UK; k Department of Endocrinology and Diabetes, University College Hospital London, London NW12BU, UK; l Queen Elizabeth Hospital, Birmingham B152WB, UK; m Department of Diagnostic and Interventional Radiology, Jena University Hospital, Jena 07747, Germany

**Keywords:** Androgens, Congenital Adrenal Hyperplasia, CAH, Gray matter, Magnetic resonance imaging, MRI, Voxel-based morphometry, VBM

## Abstract

Congenital Adrenal Hyperplasia (CAH) has been reported to present with gray matter aberrations, but further research is required as the results of existing studies are inconsistent. These inconsistences might be due to small sample sizes, differences in sample composition (some studies only included females), or the spatial scale of the analyses (some studies focused on selected regions of interest). Here we compiled the largest CAH sample to date comprising 33 women and 20 men matched to 33 control women and 20 control men on sex, age, education, and verbal intelligence. Gray matter was examined with a voxel-wise regional specificity across the entire brain, assessing the main effects of CAH and sex, and the CAH-by-sex interaction (the latter reflecting effects of prenatal androgen exposure). Individuals with CAH had significantly less gray matter compared to controls within the amygdala, calcarine cortex (specifically the primary visual area), and parahippocampal cortex (specifically the subiculum). There also was a main effect of sex, with more gray matter in females than males in medial frontal regions and more gray matter in males than females within the cerebellum. There was no CAH-by-sex interaction. Our findings indicate less regional gray matter in individuals with CAH, which seems to be caused by the medical condition itself and/or its treatment with glucocorticoids, rather than by elevated prenatal androgen exposure.

## 1. Introduction

Congenital Adrenal Hyperplasia (CAH) is a rare genetic condition resulting in compromised cortisol synthesis and concurrent high prenatal androgen levels in females but not males (Merke and Auchus, 2020; Pang et al., 1980). More specifically, as described elsewhere (Claahsen van der Grinten et al., 2022), the most common cause for CAH is a mutation in the *CYP21A2* gene, which encodes the enzyme 21-hydroxylase that is essential for synthesizing cortisol in the adrenal glands. When this enzyme is deficient, cortisol production is reduced and the precursors that would normally be used to make cortisol are instead converted into androgens. This leads to lower prenatal cortisol levels in both sexes, but elevated prenatal androgen levels only in females. In males, there is no net increase in prenatal androgens because the production of testicular androgens is automatically decreased in response to the increased adrenal-derived androgens (due to the compromised cortisol production).

After birth, cortisol is supplemented as a life-long treatment in both females and males, which reduces excessive androgen levels in females (Merke and Auchus, 2020). Several neuroimaging studies have investigated the effects of CAH on the brain, frequently to gain insights into the effects of prenatal androgen exposure (for review see Beltz et al., 2021; Khalifeh et al., 2022). While these studies observed structural differences associated with CAH (Khalifeh et al., 2022), findings lack consistency in terms of the specific region(s) affected. For example, with respect to gray matter (i.e., one of the brain’s main tissue types), significant differences between individuals with CAH and controls emerged in the left hemisphere or right hemisphere; in cortical or subcortical regions; and within the frontal, temporal, parietal, or occipital lobes (Beltz et al., 2021; Khalifeh et al., 2022). Such varied findings might be due to small sample sizes (CAH is a rare condition), differences in sample composition (some studies only included females), differences in the applied morphometric approach (some studies restricted their analysis to regions of interest).

Therefore, to gain additional insights into gray matter aberrations related to CAH, we assessed the largest CAH sample to date (n = 106). We included both women (33 CAH / 33 controls) and men (20 CAH / 20 controls) and used voxel-based morphometry (VBM). This morpho- metric method allows detection of group differences across the entire brain with a high regional specificity (voxel by voxel) without requiring the a priori definition of particular regions of interest (Kurth et al., 2015). Based on previous reports, we expected less gray matter in CAH compared to controls but had no particular hypothesis in terms of the specific brain region(s) affected. In addition, we investigated sex differences and CAH-by-sex interactions; the latter would reflect the effects of prenatal androgen exposure if the effects were detected only in females (Beltz et al., 2021).

## 2. Methods

### 2.1. Study sample

Study participants were recruited in the United Kingdom through National Health Service (NHS) clinics, a national CAH support group, as well as flyers and advertisements posted in hospitals, general practice clinics, and online. The study sample included 53 individuals (33 women and 20 men) with classic CAH,^1^ all with 21-hydroxylase deficiency, aged between 18 and 46 years (mean ± SD: 30.15 ± 7.92 years), and 53 controls (33 women and 20 men), aged between 18 and 45 years (mean ± SD: 30.34 ± 7.71 years). Individuals with CAH were pair-wise matched to controls with respect to sex, age, education, and verbal intelligence. All participants were free from neurological or psychiatric disorders and had no contraindications to magnetic resonance imaging (MRI). The study was approved by a National Health Services Research Ethics Committee and the Health Research Authority in the United Kingdom (15/EM/0532) as well as the Ethics Committee at the University of Auckland in New Zealand (020825). All participants provided their informed consent.

### 2.2. Image data acquisition

Structural T1-weighted images of the brain were acquired from each participant on a Siemens 3.0 Tesla Skyra system with a 32-channel head coil using the following parameters: TR = 2300 ms, TE = 2.98 ms, flip angle = 9°, matrix size = 256 x 240, 176 sagittal sections, voxel size = 1 × 1 × 1 mm^3^.

### 2.3. Image data processing

All brain images were processed via the CAT12 (version 12.8; Gaser et al., 2024) and SPM12 (version r7771; https://www.fil.ion.ucl.ac.uk/spm/) using VBM, as described elsewhere (Ashburner and Friston, 2000; Kurth et al., 2015). Briefly, images were corrected for magnetic field inhomogeneities, classified as gray matter (GM), white matter (WM) and cerebrospinal fluid (CSF), and spatially normalized at a resolution of 1.5 × 1.5 × 1.5 mm^3^ using linear transformations and non-linear warping (Ashburner and Friston, 2011). The normalized GM segments were then modulated by the Jacobian determinant derived from the normalization matrix to preserve the original voxel-wise GM (Ashburner and Friston, 2000; Kurth et al., 2015). The resulting modulated normalized GM segments were smoothed using a 4 × 4 × 4 mm^3^ Gaussian kernel. In addition, the total intracranial volume (TIV) was estimated (in ml) by adding the tissue volumes of GM, WM, and CSF.

### 2.4. Statistical analysis

The statistical analysis was performed using the general linear model as implemented in SPM to assess the effects of CAH (CAH vs. controls), sex (female vs. male), and any CAH-by-sex interaction. The dependent variables were the smoothed modulated normalized GM segments. The independent variables were CAH, sex, and the CAH-by-sex interaction; and the covariates were TIV and age.^2^ Significance was established using non-parametric statistics based on the threshold-free cluster enhancement (TFCE; Smith and Nichols, 2009), as implemented in the TFCE toolbox (r269; https://neuro.uni-jena.github.io/software). The significance maps were thresholded at p < 0.05 after controlling for the family-wise error (FWE) rate. Finally, the Neuromorphometrics Atlas (http://Neuromorphometrics.com) was used to determine the underlying anatomical region pertaining to the local maximum of each significance cluster.

## 3. Results

As shown in Table 1 and Supplemental Fig. 1, there was a significant main effect of CAH, where individuals with CAH had significantly less gray matter than controls. More specifically, this effect was observed within the (1) right amygdala, (2) left amygdala, (3) bilateral calcarine cortex (primary visual area), (4) left parahippocampal cortex (sub- iculum) and (5) left lingual gyrus. There was no cluster where individuals with CAH had significantly more gray matter than controls.

As shown in Table 2 and Supplemental Fig. 2, there was a significant main effect of sex in both directions: Females had significantly more gray matter than males in two clusters located within the (1) right medial frontal and anterior cingulate cortex and the (2) left medial precentral gyrus. Males, on the other hand, had significantly more gray matter than females within the cerebellum (specifically Crus I) in two separate clusters (labeled as cluster 3 and cluster 4 in Table 2). There was no significant CAH-by-sex interaction.

## 4. Discussion

Classic CAH has been linked to elevated androgen exposure in female fetuses, whereas androgen levels in male fetuses are largely unchanged. If fetal androgens were a key contributor to gray matter alterations in CAH, there would be a significant CAH-by-sex interaction (i.e., different effects when comparing women with CAH to control women and when comparing men with CAH to control men). However, we did not find a significant CAH-by-sex interaction. Instead, we observed significant main effects of CAH and significant main effects of sex. The focus of the current paper is on CAH, so the remainder of the discussion will primarily address the CAH effect. Nevertheless, it is worth noting that the locations of the significant sex effects agree with prior reports from other VBM studies (e.g., Goldstein et al., 2001; Lotze et al., 2019).

The observed main effect of CAH suggests that regional variations in gray matter might be linked to aspects of the condition itself and/or its treatment. For example, both women and men with CAH receive supplements of glucocorticoids which may lead to elevated or abnormal glucocorticoid levels. These, in turn, may cause changes in dendritic morphology as well as shrinkage or even loss of pyramidal cells within the hippocampus (Woolley et al., 1990), a brain region that contains a high concentration of corticosteroid-binding sites. Notably, even outside the framework of CAH, both animal and human studies suggest hippo- campal atrophy following prolonged exposure to glucocorticoid excess (Sapolsky et al., 1990; Starkman et al., 1992).

Our finding for the hippocampal subiculum is consistent with other reports of reduced volumes within the hippocampus, including the subiculum, in individuals with CAH (Beltz et al., 2021; Herting et al., 2020; Khalifeh et al., 2022; Webb et al., 2018). However, one of these studies reported effects in the right but not the left hippocampus (Webb et al., 2018) and other studies which investigated the hippocampus in particular (among other brain regions) did not detect any significant difference between individuals with CAH and controls (Merke et al., 2003; Van’t Westeinde et al., 2020). With respect to the amygdala clusters, our findings agree with studies reporting smaller volumes in people with CAH than in controls (Herting et al., 2020; Merke et al., 2003; Rose et al., 2004), but disagree with others who reported a lack of such group differences (Mnif et al., 2012; Webb et al., 2018). Finally, with respect to the calcarine gyrus cluster, one study (Van’t Westeinde et al., 2020) observed smaller gray matter volumes in the left pericalcarine cortex when contrasting individuals with CAH who were treated prenatally with dexamethasone to individuals with CAH that were not treated prenatally. However, when combining both CAH groups and contrasting them to controls, the same study (Van’t Westeinde et al., 2020) detected an increased surface area of the pericalcarine cortex in patients with CAH.

In conclusion, the current findings suggest that gray matter variations in CAH may result from the condition itself and, potentially, its treatment with glucocorticoids, rather than fetal androgen exposure. Further exploration of the underlying mechanisms, including the different effects of glucocorticoid treatment, would be of value. Thus, follow-up studies relating individual cortisol levels and other aspects of treatment (information that was not available for the current cohort) are indicated. Last but not least, we would like to emphasize that the current study was conducted in a sample of 106 individuals. Thus, the analyses might have been underpowered to detect a significant CAH-by-sex interaction, especially if effects are only small or medium. Classic CAH affects approximately 1 in 16,000 births (Speiser and White, 2003). This makes it extremely challenging to recruit participants (the current CAH sample is the largest to date). However, future research would immensely benefit from increasing the size of the CAH sample to allow for more definitive conclusions with respect to CAH-by-sex interactions and the possible (enduring) impact of fetal androgens on local gray matter.

## Supplementary Material

1

## Figures and Tables

**Table 1:** Significant main effects of CAH.

	Cluster region (x; y; z)^a^	Significance (p)^b^	Cluster size
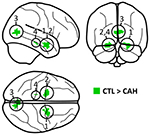	1. Right Amygdala		308 voxels
	27; 0; −26	0.018	
	16; 0; −16	0.032	
	14; −8; −18	0.034	
	2. Left Amygdala		322 voxels
	-22; −4; −24	0.026	
	3. Bilateral Calcarine Cortex		309 voxels
	−3; −74; 9	0.022	
	9; −76; 14	0.025	
	6; −81; 8	0.039	
	4. Left Parahippocampal Cortex		14 voxels
	−20; −27; −20	0.044	
	5. Left Lingual Gyrus^c^		1 voxel
	−6; 81; 12	0.050	

a Local significance maxima.

b All p-values are FWE-corrected for multiple comparisons.

c Cluster 5 only contained 1 voxel and thus is omitted on the glass brain.

**Table 2:** Significant main effects of sex.

	Cluster region (x; y; z)^a^	Significance (p)^b^	Cluster size
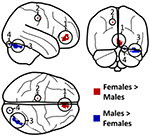	1. Right Medial Frontal / Anterior Cingulate Cortex		221 voxels
	9; 38; −10	p = 0.031	
	0; 44; 3	p = 0.032	
	8; 46; −6	p = 0.035	
	2. Left Medial Precentral Gyrus		9 voxels
	−12; −24; 45	p = 0.043	
	3. Right Cerebellum		237 voxels
	39; −78; −20	p = 0.024	
	44; −72; −22	p = 0.026	
	50; −63; −26	p = 0.027	
	4. Right Cerebellum		12 voxels
	15; −88; −20	p = 0.044	

a Local significance maxima.

b All p-values are FWE-corrected for multiple comparisons.

## Footnotes

1 Of the 53 individuals with CAH, 29 presented with a salt-wasting phenotype (20 women, 9 men) and 18 with a simple virilizing phenotype (10 women, 8 men); the remaining 6 individuals with CAH did not have information on the form of the condition.

2 Supplemental analyses were conducted without co-varying for TIV; the outcomes are provided in Supplemental Table 1. In addition, supplemental analyses were conducted to map the link between voxel-wise gray matter and TIV; the outcomes are provided in Supplemental Fig. 3.

## Appendix A. Supplementary data

Supplementary data to this article can be found online at https://doi.org/10.1016/j.yhbeh.2025.105766.
