# Supplementary material for: Altered regional gray matter in Congenital Adrenal Hyperplasia (CAH)
Source: Horm Behav. Author manuscript; Available in PMC 2026 Jun 3. (PMC13231460; doi:10.1016/j.yhbeh.2025.105766)
Supplement: 1 [file NIHMS2176715-supplement-1.docx]

**Supplemental Material**


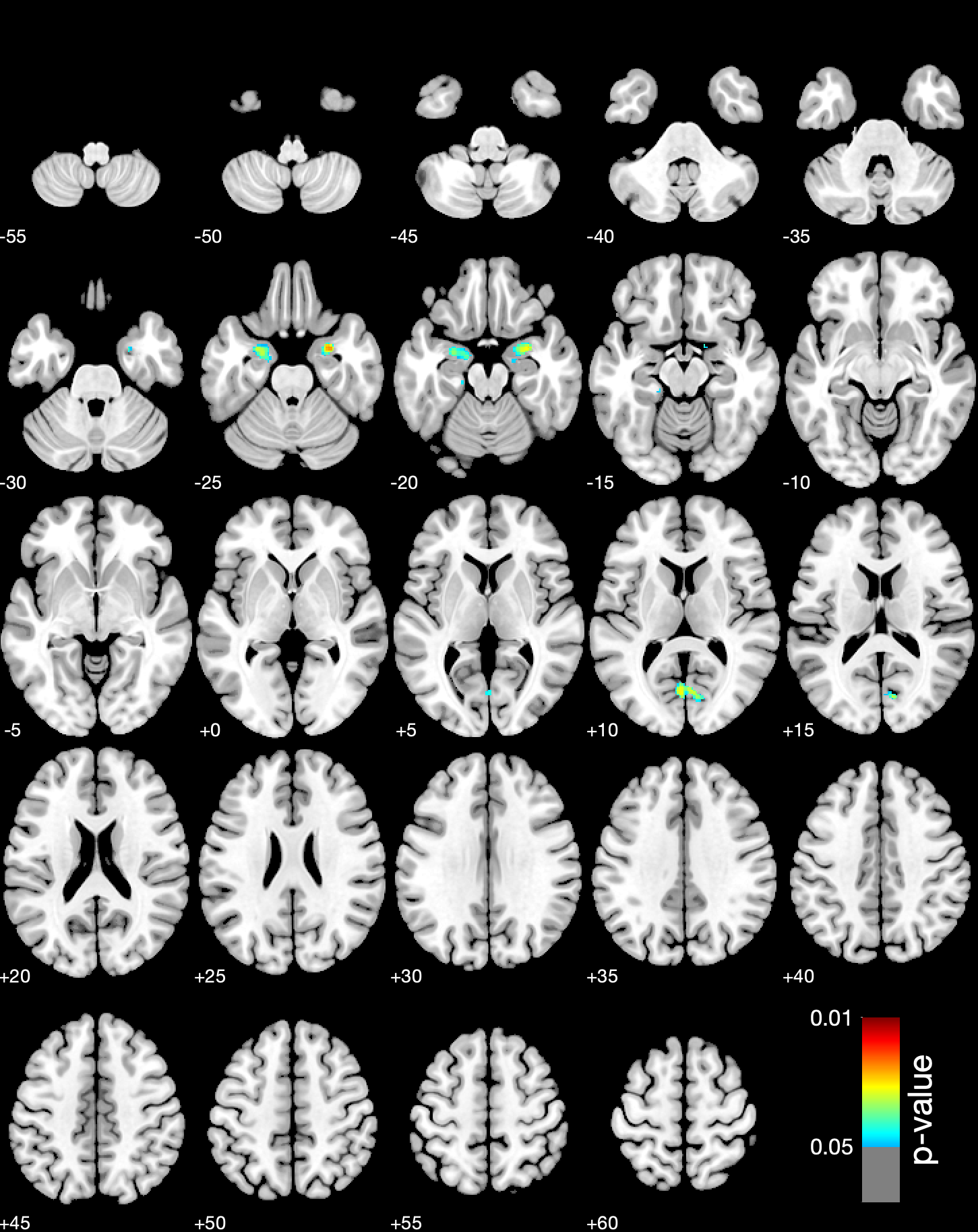


**Supplemental Figure 1**. Significant main effects of CAH. Less gray matter in individuals with CAH compared to controls. Significance clusters are projected onto axial sections of the *MNI152NLin2009cAsym* template. The color bar encodes significance (p), corrected for multiple comparisons. There was no region where individuals with CAH had more gray matter than controls.


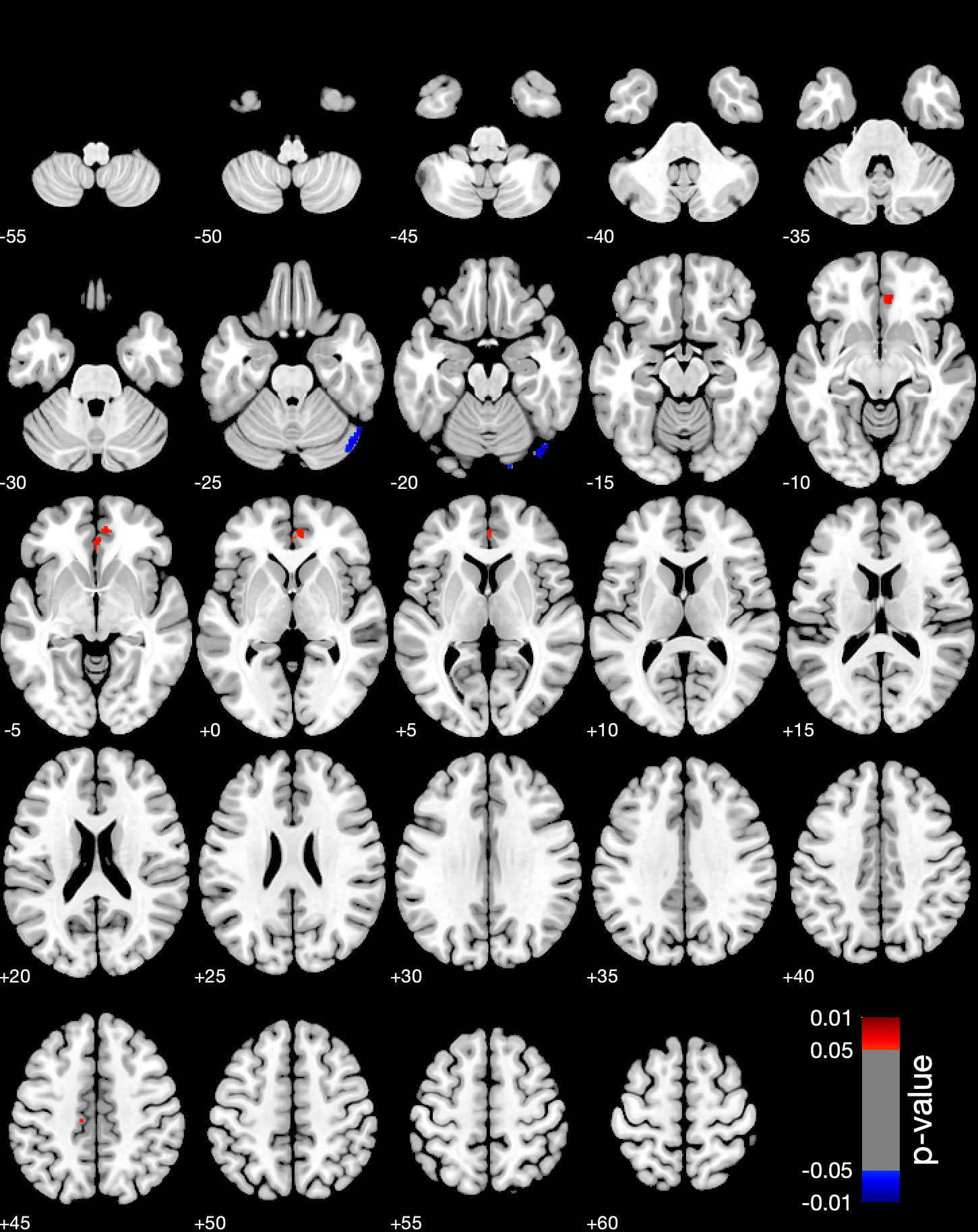


**Supplemental Figure 2.** Significant main effects of sex. More gray matter in females compared to males (red clusters) as well as more gray matter in males compared to females (blue clusters). Significance clusters are projected onto axial sections of the *MNI152NLin2009cAsym* template. The color bar encodes significance (p), corrected for multiple comparisons.

**Supplementary Table 1.** Significant main effects of CAH without co-varying for TIV

| 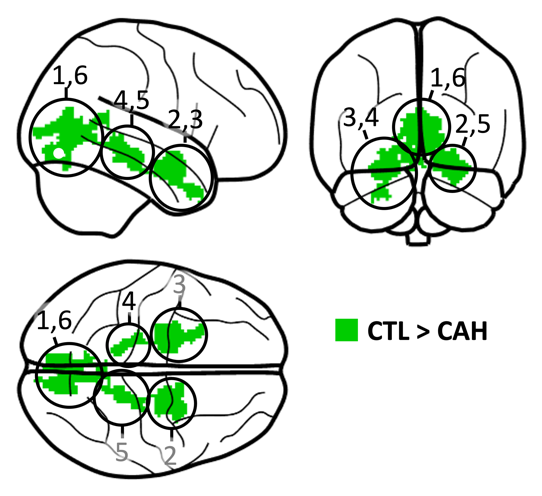 | **Cluster Region**  **(x; y; z)*** | **Significance**  **(p)**** | **Cluster Size** |
| --- | --- | --- | --- |
|  | 1. Bilateral Calcarine Cortex  -3 -74 9  9 -76 14  6 -81 8 | 0.019  0.02  0.024 | 1,960 voxels |
|  | 2. Right Amygdala and right Hippocampus  27 0 -27  16 0 -16  18 -12 -18 | 0.021  0.025  0.028 | 1,113 voxels |
|  | 3. Left Amygdala, left Hippocampus and left Temporal pole  -22 -3 -24  -15 -9 -18  -27 15 -39 | 0.023  0.026  0.038 | 1,154 voxels |
|  | 4. Left Hippocampus and left Parahippocampal Cortex  -18 -32 -15  -26 -27 -9  -12 -39 -4 | 0.027  0.043  0.047 | 259 voxels |
|  | 5. Right Lingual Gyrus and right Parahipocampal Cortex  16 -39 -10  18 -28 -20  14 -46 -4 | 0.038  0.039  0.042 | 381 voxel |
|  | 6. Right Lingual Gyrus  14 -70 -6 | 0.046 | 12 voxel |

* Local significance maxima

** All p-values are FWE-corrected for multiple comparisons.


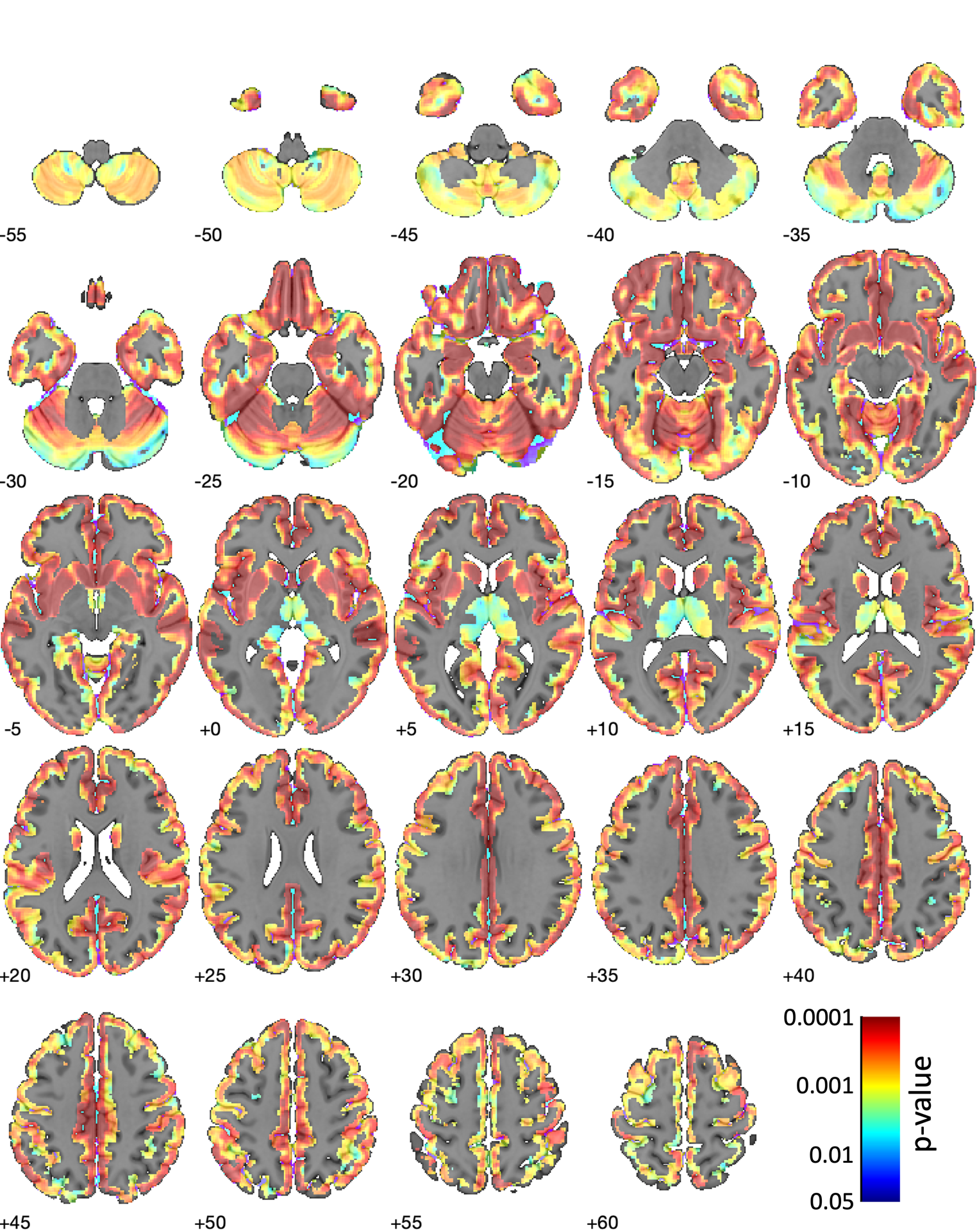


**Supplemental Figure 3.** Significant increase in gray matter volume with increasing TIV. Significance clusters are projected onto axial sections of the *MNI152NLin2009cAsym* template. The color bar encodes significance (p), corrected for multiple comparisons.
